# 3‐Cl‐AHPC inhibits pro‐HGF maturation by inducing matriptase/HAI‐1 complex formation

**DOI:** 10.1111/jcmm.13900

**Published:** 2018-10-28

**Authors:** Fang Ye, Shuang Chen, Xingxing Liu, Xiaohong Ye, Keqi Wang, Zhiping Zeng, Ying Su, Xiao‐kun Zhang, Hu Zhou

**Affiliations:** ^1^ School of Pharmaceutical Sciences Fujian Provincial Key Laboratory of Innovative Drug Target Research Xiamen University Xiamen Fujian China; ^2^ Cancer Center Sanford Burnham Prebys Medical Discovery Institute La Jolla CA USA

**Keywords:** 3‐Cl‐AHPC, complex, HAI‐1, HGF, matriptase, pro‐HGF, RARγ

## Abstract

Matriptase is an epithelia‐specific membrane‐anchored serine protease, and its dysregulation is highly related to the progression of a variety of cancers. Hepatocyte growth factor activator inhibitor‐1 (HAI‐1) inhibits matriptase activity through forming complex with activated matriptase. The balance of matriptase activation and matriptase/HAI‐1 complex formation determines the intensity and duration of matriptase activity. 3‐Cl‐AHPC, 4‐[3‐(1‐adamantyl)‐4‐hydroxyphenyl]‐3‐chlorocinnamic acid, is an adamantly substituted retinoid‐related molecule and a ligand of retinoic acid receptor γ (RARγ). 3‐Cl‐AHPC is of strong anti‐cancer effect but with elusive mechanisms. In our current study, we show that 3‐Cl‐AHPC time‐ and dose‐ dependently induces matriptase/HAI‐1 complex formation, leading to the suppression of activated matriptase in cancer cells and tissues. Furthermore, 3‐Cl‐AHPC promotes matriptase shedding but without increasing the activity of shed matriptase. Moreover, 3‐Cl‐AHPC inhibits matriptase‐mediated cleavage of pro‐HGF through matriptase/HAI‐1 complex induction, resulting in the suppression of pro‐HGF‐stimulated signalling and cell scattering. Although 3‐Cl‐AHPC binds to RARγ, its induction of matriptase/HAI‐1 complex is not RARγ dependent. Together, our data demonstrates that 3‐Cl‐AHPC down‐regulates matriptase activity through induction of matriptase/HAI‐1 complex formation in a RARγ‐independent manner, providing a mechanism of 3‐Cl‐AHPC anti‐cancer activity and a new strategy to inhibit abnormal matriptase activity via matriptase/HAI‐1 complex induction using small molecules.

## 1 INTRODUCTION

Matriptase is a type II transmembrane serine protease that plays an essential role in neonatal and post‐natal development, tissue homoeostasis, as well as tumour progression.^1^, ^2^ Matriptase is first synthesized as an inactivate zymogen that undergoes autoactivation through two sequential cleavage processes, of which the first and second cleavages occur after Gly149 and Arg614 respectively.^1^ Activated matriptase catalyzes the cleavage of a variety of substrates including prostansin, G‐protein‐coupled protease activated receptor‐2, platelet‐derived growth factor‐D, urokinase plasminogen activator and hepatocyte growth factor (HGF).^2^, ^3^ It has been elucidated that the expression and activation of matriptase highly correlates with the progression of breast, prostate, ovary, uterus, cervix, colon and skin cancers,^2^, ^3^, ^4^ indicating that up‐regulated matriptase proteolytic activity is a common cause of cancer progression. Matriptase autoactivation is greatly increased by an acidic environment,^5^ which may explain the high activation of matriptase in solid tumours with a mildly acidic extracellular microenvironment (ECM). Several oncogenic agents and signals including epidermal growth factor (EGF), androgen and ErbB‐2 potently activate matriptase in cancer cells and tissues.^6^, ^7^, ^8^, ^9^ Therefore, matriptase is a potent oncogenic protein, making it a potential drug target for cancer therapy.

One of the oncogenic actions of matriptase is to activate HGF.^10^, ^11^, ^12^ Cell first synthesizes an inactive single chain pro‐HGF secreted to ECM.^13^, ^14^, ^15^ Pro‐HGF is able to bind to but unable to activate its membrane surface receptor c‐Met.^16^ Activated matriptase catalyzes the cleavage of pro‐HGF to produce mature HGF containing α and β chains.^10^, ^11^, ^12^ HGF is a pleiotropic growth factor with strong stimulations of cell migration, proliferation, survival and morphogenesis. HGF activation by matriptase and the subsequent induction of c‐Met pathway contribute to the progression of cancers.^17^, ^18^, ^19^ Thus, a rational strategy to suppress HGF activity in cancers is to inhibit its matriptase‐mediated maturation from pro‐HGF.

HGF activator inhibitor type‐1 (HAI‐1) is a cognate partner of activated matriptase.^20^ Mice embryogenesis perish as a result of HAI‐1 deletions is reversed in matriptase/HAI‐1 double‐deficient mice,^21^, ^22^ whereas ectopically expressed HAI‐1 antagonizes the oncogenic properties of matriptase in mice.^23^ Thus, HAI‐1 is an endogenous inhibitor of matriptase. Upon activation, matriptase is complexed by HAI‐1, which largely quenches matriptase proteolytic activity. This is a major approach for cell to avoid excess and uncontrolled activity of matriptase.^1^, ^20^ The ratio of matriptase/HAI‐1 is generally much higher in cancer cells than in normal cells,^24^, ^25^, ^26^ rendering the high activating level of matriptase in cancer cells. Thus, the increase of HAI‐I and/or the formation of matriptase/HAI‐1 complex should inhibit matriptase activity in cancers.

The high correlation of matriptase activity and cancer progression intrigues the studies of targeting matriptase for cancer treatment. Indeed, some natural and synthetic agents such as curcumin inhibit matriptase activity to exert potent anti‐cancer efficacy.^6^, ^27^, ^28^, ^29^ 3‐Cl‐AHPC, 4‐[3‐(1‐adamantyl)‐4‐hydroxyphenyl]‐3‐chlorocinnamic acid, is a selective ligand of retinoic acid receptor γ (RARγ) and has strong anti‐tumour effects in both RARγ‐dependent and ‐independent manners.^30^ The anti‐cancer effect of 3‐Cl‐AHPC relies on its inhibition of tumour growth and migration, which has been widely established although the underlying mechanisms remain elusive.^31^, ^32^, ^33^ Here, we unravelled a novel mechanism of 3‐Cl‐AHPC anti‐cancer effect by inducing matriptase/HAI‐1 complex formation to inhibit matriptase proteolytic activity in a RARγ‐independent manner.

## 2 MATERIALS AND METHODS

### 2.1 Reagents

Dulbecco's Modified Eagle Medium (DMEM) and RPMI1640 medium were obtained from Hyclone (Logan, UT, USA). DMEM‐F‐12 were obtained from Gibco (Paisley, Scotland, UK). Protein Assay kits were from Thermo Fisher Scientific (Rockford, IL, USA). LipofectamineTM 2000 reagent were purchased from Invitrogen. M24, M69 and M19 antibodies were gifts kindly from Dr. Chen‐Yong Lin at Georgetown University, Washington, D.C., United States. Antibodies against β‐actin (A5441) and Flag (F1804) and siRNAs for matriptase (NM_021978) and RARγ (NM_000966) were purchased from Sigma‐Aldrich (St. Louis, MO, USA). Antibodies against c‐Met (8198P), p‐Met (Tyr‐1234/1235) (#3077), Gab1 (3232S), p‐Gab1 (3231) were purchased from Cell Signaling Technology (Beverly, MA, USA). Antibodies against RARγ (C‐19, sc‐550), Matriptase (D‐7, sc‐365482) were purchased from Santa Cruz Biology (Santa Cruz, CA, USA). The goat antimouse (31436) and anti‐rabbit (31461) IgG F(ab’) secondary antibodies were purchased from Thermo Fisher Scientific. pro‐HGF(7057‐HG) was from R&D Systems (Minneapolis, MN, USA). Boc‐Gln‐Ala‐Arg‐AMC (BML‐P237‐0005) was purchased from Enzo Life Sciences (Farmingdale, NY, USA).

### 2.2 Cell culture

A431, MCF‐7, MCF‐10A, SW620 and HCT116 cells were obtained from American Type Culture Collection (ATCC). A431, SW620 and MCF‐7 cells were maintained in DMEM containing 10% foetal bovine serum (FBS). HCT116 cells were cultured in RPMI‐1640 supplemented with 10% FBS. MCF‐10A cells were cultured in DMEM‐F‐12 supplemented with 10% FBS. All cell lines were authenticated by ATCC using morphology, karyotyping and PCR‐based approaches. All cell lines were used in less than 6 months of continuous passage after acquisition.

### 2.3 Western blot analysis

The protein extracts were mixed with protein loading buffer in a non‐reducing and non‐boiling conditions for detecting matriptase, HAI‐1 and matriptase/HAI‐1 complex using M24, M19 and M69 antibodies. For other protein detections, the protein extracts were boiled in sodium dodecyl sulphate (SDS) sample loading buffer containing 5% β‐mercaptoethanol. Samples were resolved by 8% SDS‐polyacrylamide gel electrophoresis (SDS‐PAGE) and transferred to nitrocellulose. The membranes were blocked with 5% milk in Tris‐buffered saline and tween 20 (TBST) at room temperature for 1 hour and then incubated with primary antibodies at 4°C overnight. After washed three times by TBST, membranes incubated with secondary antibody for 1 hour at room temperature followed by three washed with TBST. The protein signals were visualized using enhanced chemiluminescence reagents.

### 2.4 Real‐time polymerase chain reaction assay

Total RNA was extracted from cells with Trizol (life). A total of 2 μg RNA was used to prepare cDNA using oligo(dT) as a primer. TIANScript RT Kit's was used for real‐time polymerase chain reaction (PCR) analysis. Each sample was run in triplicate. The relative RNA amounts were calculated with the ∆∆Ct method by ABI step one PCR instrument and normalized with an internal control, GAPDH. Primers: GAPDH forward: GAAGGTGAAGGTCGGAGTC, reverse: GAAGATGGTGATGGGATTTC; Matriptase forward: CACCTCAGTGGTGGCTTTCC, reverse: GCGTGCAGGCCAAAGCT. HAI‐1forward: CCAGACACAGGACTCTGCAA, reverse: CAGGCCAAACACA TCCTTCT.

### 2.5 Gelatin zymography

For gelatin zymography, cells were treated with 3‐Cl‐AHPC(1 μM) for 12 hours. The supernatants of the conditioned media were collected and concentrated using Amicon Ultra‐4 centrifuge filter devices (Millipore, Tullagreen, Carrigtwohill Co Cork, Ireland) at 2000 *g* at 4°C for 30 minutes. Gelatin zymography was carried out on 8% polyacrylamide gels, containing 1 mg/mL gelatin. After conducting SDS‐PAGE under non‐reducing conditions, proteins separated on the gels were renatured by incubating the gels in 50 mM Tris‐HCl buffer (pH 7.5) containing 100 mM NaCl and 2.5% Triton X‐100 at room temperature for 1.5 hours and then incubated in a reaction buffer consisting of 50 mM Tris‐HCl (pH 7.5) and 5 mM CaCl_2_ at 37°C for 16 hours. The resultant gels were stained with Coomassie Brilliant Blue R‐250. To eliminate metalloproteinase activities, the renatured gels were incubated in 50 mM Tris‐HCl (pH 7.5) buffer containing 0.5 mM EDTA for 30 minutes before the reaction.

### 2.6 Cell scattering assay

A431 cells were cultured in 12‐well tissue culture plates. After colonies formed (4‐8 days), cells were serum‐starved overnight and were then treated with pro‐HGF (20 ng/mL; R&D Systems) in the presence or absence of 3‐Cl‐AHPC (0.5 μM). Images of migrating cells were captured at 48 hours after the treatment for 48 hours.

### 2.7 Wound healing/scratch assay

A431 cells were seeded in 12‐well plates and allowed to reach confluence. A scratch/wound was introduced into the cell monolayer with a sterile tip. Cells were cultured in serum‐free media or were treated with pro‐HGF (20 ng/mL) in the presence or absence of 3‐Cl‐AHPC (0.5 μM). Images of migrating cells were captured at 48 hours after the treatment.

### 2.8 Trans‐well invasion assay

Trans‐wells were coated with 20 μg of matrigel (BD Biosciences, Bedford, MA, USA) for cell invasion assay. A431 cells were then seeded in the upper chambers of trans‐wells with serum‐free medium. The lower chambers were filled with the medium containing 5% FBS, pro‐HGF and/or 3‐Cl‐AHPC (0.5 μM) as chemoattractants. After 24‐hour incubation, cells were fixed and stained with 0.1% crystal violet for 20 minutes. The penetrating cells were photographed and counted using a light microscope.

### 2.9 Proteolytic cleavage of pro‐HGF

A431 cells were serum‐starved overnight and were then treated with 3‐Cl‐AHPC (1 μM) for 12 hours. Matriptase protein extracted by using Plasma Membrane Protein Isolation Kit (cat. SM‐005, invent) incubated with pro‐HGF (50 ng) for 1 hour at 37°C. The reaction was stopped by SDS‐PAGE gel sample buffer and samples were boiled and separated by 10% PAGE. Proteins were transferred onto nitrocellulose membrane, blocked with 5% milk and immunoblotted using anti‐HGF α chain antibody (GTX129003) that recognizes pro‐HGF as well as α chain of activated HGF.

### 2.10 Protease activity assay

Cancer cells were serum‐starved overnight and were then treated with 3‐Cl‐AHPC (1 μM) for 12 hours. Cell lysate and condition medium was assessed by a fluorogenic assay measuring 7‐Amino‐4‐methylcoumarin (AMC) release from synthetic substrates by the proteases. The assay was conducted in a total volume of 200 μL which contained 5 μL of the concentrated samples, 5 μL of a 5 mM stock of the substrate (Boc‐Gln‐Ala‐Arg‐AMC) and 190 μL of 100 mM Tris HCl (pH 8.5) containing 100 μg/mL bovine serum albumin. The released fluorescence resulting from hydrolysis of the peptide substrates was measured using a fluorescent spectrophotometer (GloMax^®^ Discover Multimode Microplate Reader, Madison, WI, USA) with excitation at 360 nm and emission at 480 nm.

### 2.11 Tumour xenografts

For xenograft study, 4‐week‐old male nude mice were inoculated subcutaneously into the dorsal flank with 1 × 10^6^ A431 cells. After 10 days, mice were randomly assigned into two groups (6 mice/group): one group receiving 1 mg/kg of 3‐Cl‐AHPC and the other receiving physiological saline solution by daily intraperitoneal injection. The tumour volume and body weight of each mouse was monitored weekly. After 20 days treatment, mice were sacrificed and individual tumours were taken and weighted, and tumour tissues were used for Western blot analysis and protease activity assay.

### 2.12 Lentiviral particle preparation and infection for small hairpin RNA

Matriptase small hairpin RNA (sh matriptase, clone ID: TRCN 0000038053) or a negative control shRNA for knockdown of luciferase (sh Luc) were all in the pLKO.1‐puro vector and were packaged into lentiviral particles by using 293T cells by cotransfection with pMDL, VSVG and REV plasmids using Lipofectamine 2000. Conditioned medium from the transfected cells containing lentiviral particles was collected 30 and 72 hours after the addition of fresh medium. For lentiviral infection, cells were seeded at up to 70% confluence and cultured for 24 hours. Lentiviral infection was performed by adding 30% (V/V) of lentivirus‐containing medium to the cell culture. Twenty‐four hours after infection, infected cells were selected by exposure to 5 μg/mL puromycin for 2 days.

### 2.13 Co‐immunoprecipitation assay

A431 cells were serum‐starved overnight and then treated with 3‐Cl‐AHPC (1 μM) for 12 hours. Cells were lysed in a standard lysis buffer (1% Triton X‐100 in PBS) containing protease inhibitors (MCE, HY‐K0010). Protein extracted lysates were incubated with antibodies (M69 or M19) immobilized on Protein G Agarose beads (Millipore) and rotated in a cold room overnight. The beads were washed with 1% Triton in PBS four times. The captured proteins were eluted with 0.1 M glycine buffer (pH 2.4), and immediately neutralized with 2 M Trizma baze (Sigma‐Aldrich). The eluted proteins were boiled in SDS sample loading buffer without β‐mercaptomethanol and analysed by immunoblotting.

### 2.14 Statistical analyses

All experiments were repeated at least three times. Values are given as the mean ± SE. Statistical analyses were performed with GraphPad Prism 5.0 (Student's *t* test or one‐way ANOVA analysis) and values with *P* < 0.05 were considered statistically significant.

## 3 RESULTS

### 3.1 3‐Cl‐AHPC induces the formation of matriptase/HAI‐1 complex

**Figure 1:**
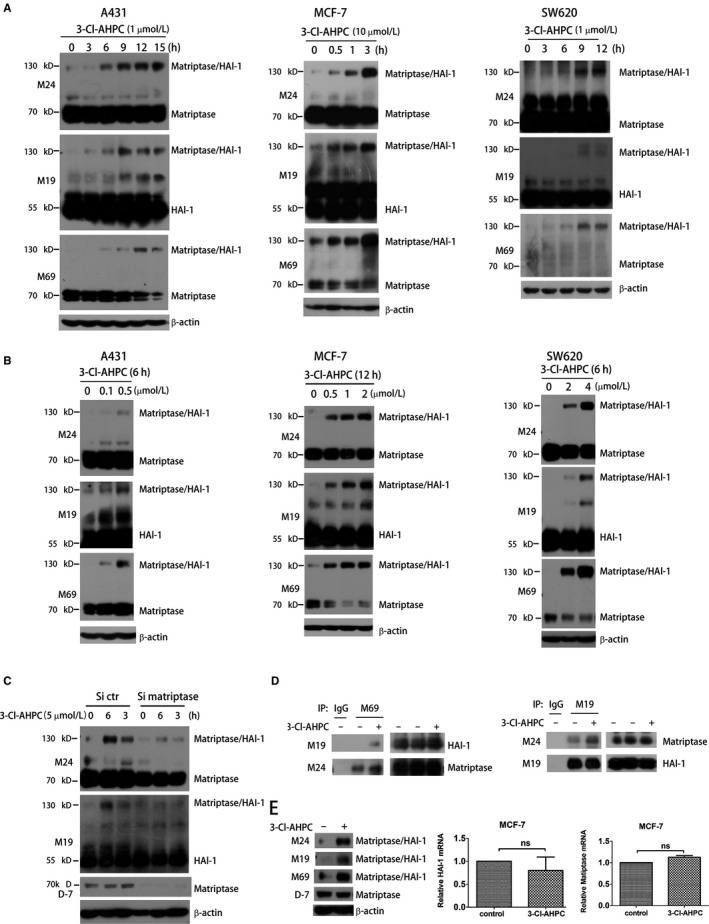
3‐Cl‐AHPC time‐ and dose‐ dependently induces matriptase/HAI‐1 complex formation. (A and B) A431, MCF‐7 and SW620 cells were treated with 1 or 10 μM 3‐Cl‐AHPC for the indicated times (A), or were treated with the indicated concentrations of 3‐Cl‐AHPC for 6 or 12 hours (B). Cell lysates were analysed by Western blotting using M24, M69 and M19 antibodies for detecting matriptase, HAI‐1 and matriptase/HAI‐1 complex. β‐actin was used as a loading control. (C) A431 cells transfected with control siRNA (Si ctr) or matriptase siRNA (Si matriptase) for 24 hours were treated with 3‐Cl‐AHPC. Cell lysates were analysed by Western blotting. (D) A431 cells were treated with 3‐Cl‐AHPC (1 μM) for 12 hours. Protein‐extracted lysates were incubated with M69 or M19 immobilized on Protein G Agarose. The protein complex was examined by Western blotting using the indicated antibodies. (E) MCF‐7 cells were treated with 5 μM 3‐Cl‐AHPC for 3 hours. Protein expressions were examined by Western blotting using the indicated antibodies, and matriptase and HAI‐1 mRNA levels were examined by qRT‐PCR (ns, no significant difference)

5α‐dihydrotestosterone (DHT), the cognate ligand of androgen receptor (AR), has been shown to activate matriptase to promote prostate cancer cell invasion and metastasis.^7^, ^8^ This prompted us to explore the effects of some natural and synthetic ligands of nuclear receptors on matriptase expression and activation. To this end, we applied Western blotting assay using anti‐total matriptase (M24), anti‐activated matriptase (M69) and anti‐HAI‐1 (M19) antibodies.^34^, ^35^ Upon activation, matriptase forms complex with HAI‐1, and all the three antibodies are able to recognize the ~120 kD complex band in the non‐denature gel. Unexpectedly, we found that 3‐Cl‐AHPC, a synthetic ligand of RARγ and a potent cancer inhibitor, strongly induced the formation of matriptase/HAI‐1 complex in breast cancer cell MCF‐7, skin cancer cell A431 and colon cancer cell SW620 (Figure 1). 3‐Cl‐AHPC at μM concentrations dramatically promoted matriptase/HAI‐1 complex formation in a time‐dependent manner (Figure 1A). When these cells were treated with increasing concentrations of 3‐Cl‐AHPC, we found 3‐Cl‐AHPC dose‐dependent induction of matriptase/HAI‐1 complex (Figure 1B). The ~120 kD band represented matriptase/HAI‐1 complex because it was not only recognized by M24 and M19 antibodies but also potently reduced by matriptase siRNA (Figure 1C). Our co‐immunoprecipitation assay also showed that 3‐Cl‐AHPC significantly induced matriptase/HAI‐1 complex formation (Figure 1D). We then explored whether the effect of 3‐Cl‐AHPC was because of its induction of matriptase and HAI‐1 expressions. D7 is an antibody that recognizes the latent matriptase. Using this antibody, we did not detect significant change of matriptase expression in MCF‐7 cells after 3‐Cl‐AHPC treatment, although the strong induction of matriptase/HAI complex was readily detected by M24, M69 and M19 antibodies (Figure 1E). In addition, our qRT‐PCR assay did not show obvious effect of 3‐Cl‐AHPC on matriptase or HAI‐1 mRNA levels (Figure 1E). Thus, 3‐Cl‐AHPC was able to induce matriptase/HAI‐1 complex formation, which was not because of the altered expression of matriptase or HAI‐1.

### 3.2 3‐Cl‐AHPC inhibits rather than activates matriptase

**Figure 2:**
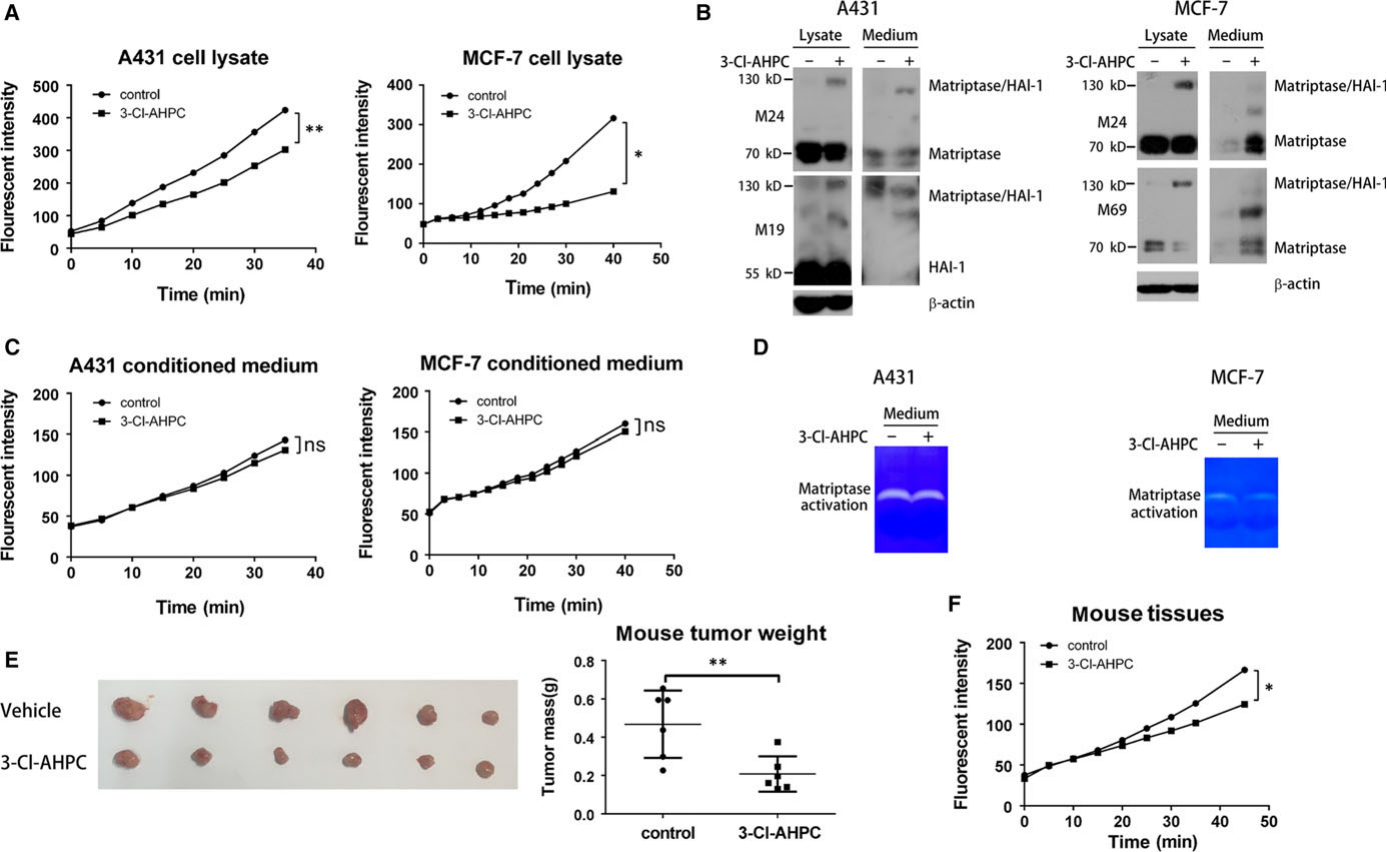
3‐Cl‐AHPC inhibits matriptase activity and induces matriptase/HAl‐1 shedding. (A‐D) A431 and MCF‐7 cells were treated with 1 μM 3‐Cl‐AHPC for 12 hours. Cell lysates (A) and the conditioned medium (C) were harvested and analysed for matriptase proteolytic activity using a matriptase synthetic fluorescent substrate Boc‐Gln‐Ala‐Arg‐AMC. Data were representative of three independent experiments under same conditions (**P < 0.01, *P < 0.05). Cell lysates and conditioned medium were analysed by Western blotting with the indicated antibodies (B). The conditioned medium was subjected to gelatin zymography assay (D). (E and F) Mice inoculated subcutaneously with A431 cells were treated with vehicle or 3‐Cl‐AHPC (1 mg/kg/day) for 20 days. The tumour mass was analysed and plotted as mean ± SE (**P < 0.01) (E). The tumour tissues were analysed for matriptase activity using a matriptase synthetic fluorescent substrate (F) (*P < 0.05)

Suramin, DHT and EGF activate matriptase followed by matriptase/HAI‐1 complex induction.^6^, ^8^, ^36^ We then investigated whether 3‐Cl‐AHPC also activated matriptase to induce the complex. We applied matriptase‐selective fluorogenic substrate to measure the proteolytic activity of matriptase.^37^ To our surprised, matriptase activity in A431 and MCF‐7 cell lysates was greatly reduced when cells were treated with 3‐Cl‐AHPC (Figure 2A). After activation and complex formation, matriptase shedding from plasma membrane initiates. When we examined cell culture medium, we found that 3‐Cl‐AHPC also enhanced matriptase shedding from A431 and MCF‐7 cells (Figure 2B). However, the proteolytic activity of shed matriptase from 3‐Cl‐AHPC‐treated cells had no increase comparing with vehicle‐treated cells in our fluorogenic substrate assay (Figure 2C) and matrigel‐enzyme assay (Figure 2D). In A431 cell‐xenografted mouse model, 3‐Cl‐AHPC dramatically inhibited tumour growth (Figure 2E). Importantly, 3‐Cl‐AHPC also inhibited matriptase activity in mouse tumour tissue (Figure 2F), implying that the anti‐matriptase activity of 3‐Cl‐AHPC contributed to its anti‐cancer efficacy. Therefore, 3‐Cl‐AHPC induced matriptase/HAI‐1 complex formation, but it inhibited rather than enhanced matriptase activity.

### 3.3 3‐Cl‐AHPC enhances HAI‐1 binding to activated matriptase

**Figure 3:**
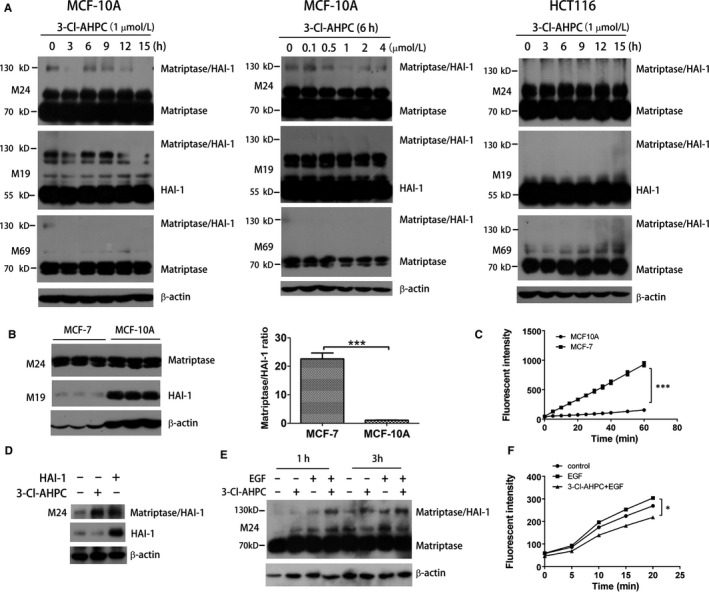
3‐Cl‐AHPC induction of matriptase/HAI‐1 complex is of cell selectivity. (A) MCF‐10A and HCT116 cells were treated with 1 μM 3‐Cl‐AHPC for the indicated times or with the indicated concentrations of 3‐Cl‐AHPC for 6 hours. Cell lysates were analysed by Western blotting using M24, M69 and M19 antibodies for matriptase, HAI‐1 and matriptase/HAI‐1 complex. (B) MCF‐7 cells and MCF‐10A cells were lysed and analysed by Western blotting for matriptase using M24 antibody and HAI‐1 using M19 antibody. The band density was quantified, and the ratio of matriptase/HAI‐1 was calculated and plotted as mean ± SE (***P < 0.001). (C) Matriptase activity in MCF‐7 and MCF‐10A cells were analysed using a matriptase synthetic fluorescent substrate (***P < 0.001). (D) A431 cells were treated with 1 μM 3‐Cl‐AHPC for 6 hours or transfected with HAI‐1 expression plasmid for 24 hours. Cell lysates were analysed by Western blotting. (E and F) Cells were treated with or without 1 μM 3‐Cl‐AHPC in the presence or absence of EGF (25 ng/mL) for 2 hours. Cell lysate were subjected for Western blotting analysis (E) or proteolytic activity assay using fluorescent substrate (F) (*P < 0.05)

Interestingly, the induction of matriptase/HAI‐1 complex by 3‐Cl‐AHPC was of cell selectivity because 3‐Cl‐AHPC failed to induce the complex in MCF‐10A and colon cancer HCT116 cells (Figure 3A). Different from MCF‐7, MCF‐10A is a non‐tumourigenic and near‐normal mammary epithelial cell line.^38^ When we compared the expression ratio of matriptase/HAI‐1 in these two cell lines, we found that MCF‐7 had much higher ratio of matriptase/HAI‐1 than MCF‐10A (Figure 3B), which was consistent with the common concept that tumour cells have higher matriptase/HAI‐1 ratio.^3^, ^4^ The higher ratio of matriptase/HAI‐1 also led to the higher proteolytic activity of matriptase in MCF‐7 cells (Figure 3C). We speculated that 3‐Cl‐AHPC could enhance the interaction between HAI‐1 and activated matriptase. In MCF‐7 cells, only part of activated matriptase formed complex with HAI‐1 because of higher ratio of matriptase/HAI‐1. On the other hand, there was still plenty of free HAI‐1 available in MCF‐7 cells. Thus, in MCF‐7 cells 3‐Cl‐AHPC induced more matriptase/HAI‐1 complex by increasing the binding affinity of HAI‐1 and activated matriptase, which was not apparent in MCF‐10A cells because majority of activated matriptase already formed complex with HAI‐1 owing to extremely low matriptase/HAI‐1 ratio as well as low matriptase activation. Similar to 3‐Cl‐AHPC treatment, ectopically overexpressed HAI‐1 in MCF‐7 cells induced more complex formation (Figure 3D). Thus, 3‐Cl‐AHPC treatment was equivalent to the increase of HAI‐1 amount. In A431 cells, 3‐Cl‐AHPC and EGF induced complex formation, respectively, and their combination induced more complex (Figure 3E). When we used fluorogenic substrate to measure the activity of matriptase, we found that EGF, as reported previously, induced matriptase proteolytic activity, which was inhibited rather than enhanced by 3‐Cl‐AHPC (Figure 3F). Thus, different from EGF activation of matriptase, 3‐Cl‐AHPC enhanced HAI‐1 binding to EGF‐activated matriptase followed by blocking matriptase activity.

### 3.4 3‐Cl‐AHPC inhibits matriptase‐mediated pro‐HGF processing

**Figure 4:**
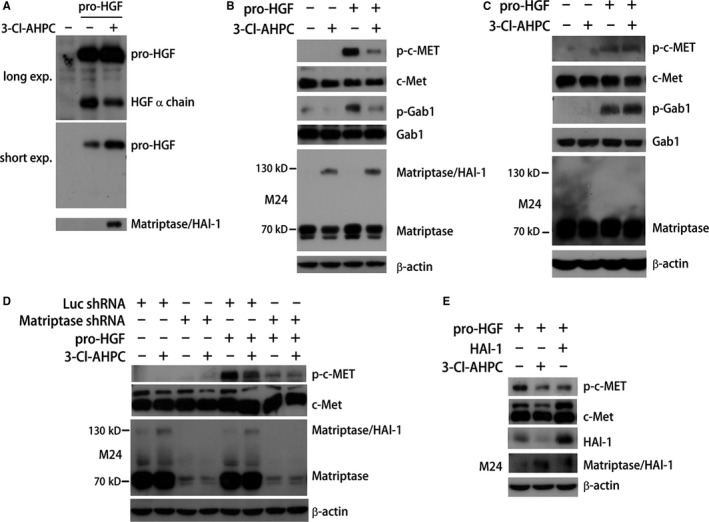
3‐Cl‐AHPC inhibits pro‐HGF maturation and signal transduction in a matriptase‐dependent manner. (A) Membrane proteins were extracted from A431 cells treated with 1 μM 3‐Cl‐AHPC for 6 hours, and then were incubated with pro‐HGF at 37°C for 1 hour. Pro‐HGF cleavage was examined by Western blotting using anti‐HGF α chain antibody. (B and C) A431 (B) or HCT116 (C) cells pre‐treated with 1 μM 3‐Cl‐AHPC for 6 hours were exposed to pro‐HGF (20 ng/mL) for 1 hour. Cell lysates were analysed by Western blotting using the indicated antibodies. (D) A431 cells were transfected with matriptase shRNA for 24 hours. Cells were then treated with 1 μM 3‐Cl‐AHPC for 6 hours before exposing to pro‐HGF. Protein expressions were examined by Western blotting using the indicated antibodies. (E) A431 cells were treated with 1 μM 3‐Cl‐AHPC for 6 hours or transfected with HAI‐1 expression plasmid for 24 hours. Cell lysates were analysed by Western blotting

Activated matriptase mediates the maturation of HGF through cleavage of pro‐HGF.^10^, ^11^ We then examined whether 3‐Cl‐AHPC inhibited matriptase‐catalyzed pro‐HGF cleavage. Purified pro‐HGF protein was incubated with cellular membrane extracts, and the mature status of pro‐HGF was determined by Western blotting assay using anti‐HGF α chain antibody. In A431 cells, 3‐Cl‐AHPC consistently induced matriptase/HAI‐1 complex formation (Figure 4A). Cellular membrane extracts from A431 cells treated with DMSO induced the cleavage of pro‐HGF, which was dramatically inhibited by 3‐Cl‐AHPC indicating by the increased pro‐HGF and the decreased HGF α chain (Figure 4A). This demonstrated that 3‐Cl‐AHPC inhibited pro‐HGF maturation, likely because of its inhibition of activated matriptase. To further confirm this in vitro effect, we examined HGF downstream signalling activity in cells. As shown in Figure 4B, pro‐HGF could strongly induce c‐Met and Gab1 phosphorylation. When cells were pre‐treated with 3‐Cl‐AHPC, the effect of pro‐HGF on activation of c‐Met and Gab1 was largely blocked accompanying with matriptase/HAI‐1 complex induction (Figure 4B). As to HCT116 cells, in which 3‐Cl‐AHPC failed to induce matriptase/HAI‐1 complex (Figures 3A and 4C), 3‐Cl‐AHPC also failed to inhibit pro‐HGF‐induced phosphorylation of c‐Met and Gab1 (Figure 4C). These results indicated the correlation of 3‐Cl‐AHPC inhibition of pro‐HGF maturation, 3‐Cl‐AHPC inhibition of pro‐HGF signal transduction and 3‐Cl‐AHPC induction of matriptase/HAI‐1 complex. The maturation of pro‐HGF was matriptase‐dependent, because pro‐HGF‐induced c‐Met activation was strongly inhibited by matriptase shRNA that largely blocked matriptase expression (Figure 4D). The suppression of matriptase expression by shRNA also abolished the inhibitory effect of 3‐Cl‐AHPC on pro‐HGF‐induced c‐Met activation, suggesting that matriptase mediated 3‐Cl‐AHPC inhibition of pro‐HGF maturation and downstream signal transduction (Figure 4D). Similar to 3‐Cl‐AHPC treatment, HAI‐1 overexpression led to the suppression of pro‐HGF‐induced c‐Met phosphorylation accompanying with matriptase/HAI‐1 complex induction (Figure 4E). Together, these results indicated that 3‐Cl‐AHPC inhibited matriptase‐mediated pro‐HGF maturation likely by inducing matriptase/HAI‐1 complex.

### 3.5 3‐Cl‐AHPC inhibits pro‐HGF‐induced cell scattering, migration and invasion

**Figure 5:**
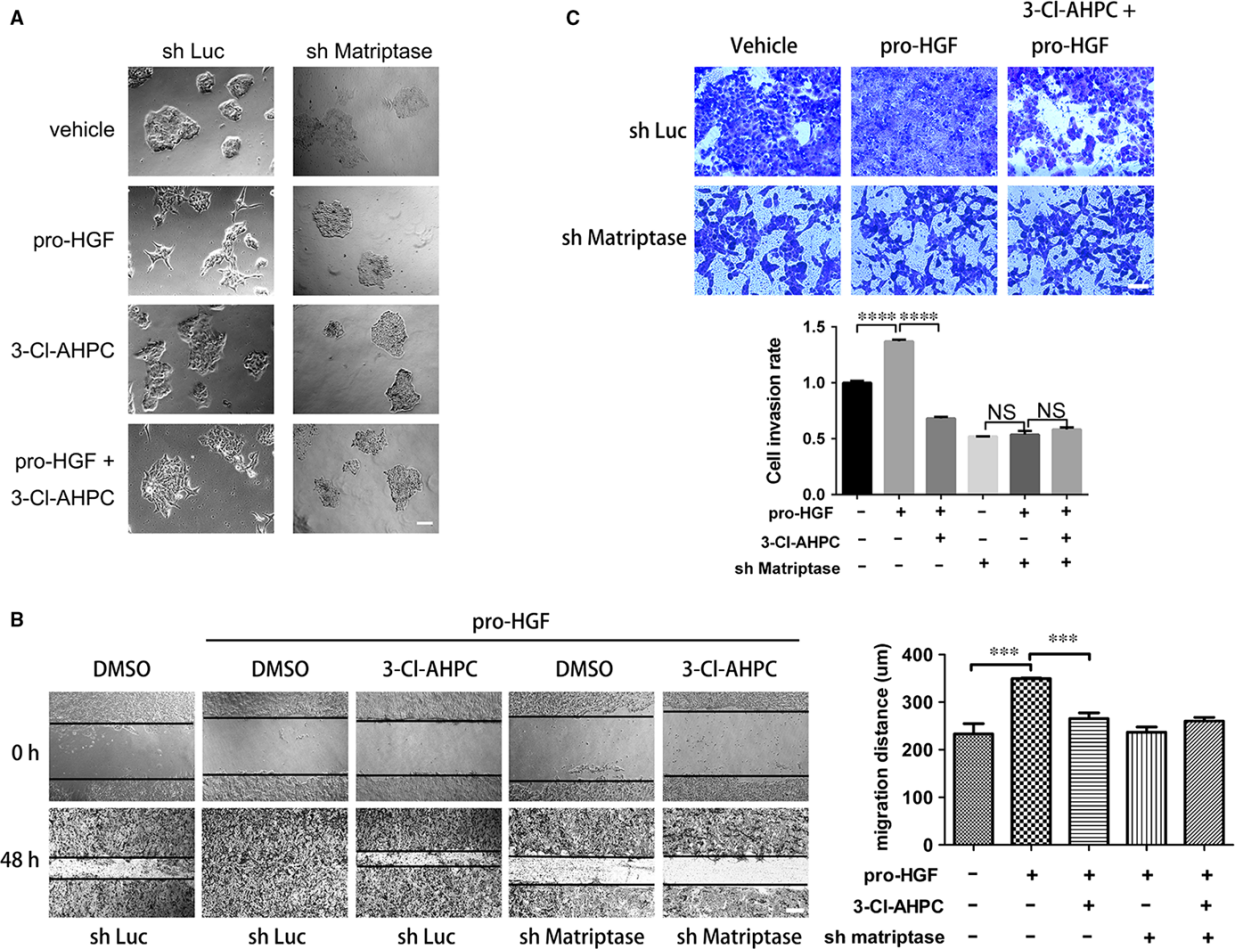
3‐Cl‐AHPC inhibits pro‐HGF‐induced cell scattering and migration. (A) Luciferase shRNA (sh Luc) and matriptase shRNA (sh matriptase) stable A431 cell lines were treated with pro‐HGF (20 ng/mL) and/or 1 μM 3‐Cl‐AHPC for 48 hours. Shown are the representative bright‐field micrographs. Scale bar represents 100 μm. (B) Pipette tips were used to make wounds with width of approximate 380 μm by scraping. Cells were then treated with pro‐HGF with or without 3‐Cl‐AHPC for 24 hours. Images were captured by a light microscopy with a magnification of 100 × , and the lines define the edges of the wounds. Migration distances were measured and plotted as mean ± SE (***P < 0.001). Scale bar represents 100 μm. (C) A431 cells were seeded in the upper chambers of trans‐wells coated with matrigel. The lower chambers were filled with the medium containing 5% FBS with or without pro‐HGF (20 ng/mL) and 3‐Cl‐AHPC (0.5 μM). After 24 hours incubation, cells were fixed and stained with 0.1% crystal violet for 20 minutes. The penetrating cells were photographed and counted (****P < 0.0001). Scale bar represents 100 μm

Hepatocyte growth factor is one of the most potent cytokines that promote cell scattering and migration.^39^, ^40^ We further characterized matriptase‐mediated 3‐Cl‐AHPC effect on pro‐HGF‐induced cell scattering. In normal culture condition, A431 cells normally formed clusters as shown in Figure 5A. When cells were treated with pro‐HGF for 24 hours, they became motile and scatter in many directions. This scattering effect disappeared once matriptase expression was repressed by shRNA, implying that matriptase on the cell surface or in the medium catalyzed the maturation of pro‐HGF (Figure 5A). When cells were treated with pro‐HGF together with 3‐Cl‐AHPC, pro‐HGF‐induced cell scattering was largely blocked (Figure 5A). Similar result was also observed in our cell scratch assay (Figure 5B). As shown in Figure 5B, pro‐HGF strongly promoted A431 cell migration in a matriptase‐dependent manner. 3‐Cl‐APHC potently inhibited pro‐HGF‐stimulated cell migration, which was also matriptase‐dependent because knockdown of matriptase disabled 3‐Cl‐AHPC (Figure 5B). Moreover, our trans‐well experiment also showed that pro‐HGF‐induced cell migration and invasion was matriptase‐dependent and largely inhibited by 3‐Cl‐AHPC (Figure 5C). Thus, 3‐Cl‐AHPC inhibited pro‐HGF‐stimulated A431 cell scattering, migration and invasion likely through inhibiting matriptase‐mediated pro‐HGF maturation.

### 3.6 3‐Cl‐AHPC induction of matriptase/HAI‐1 complex formation is not RARγ dependent

**Figure 6:**
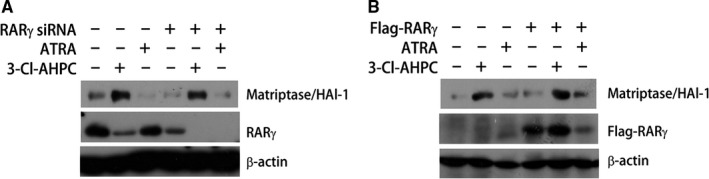
The induction of matriptase/HAl‐1 complex formation is not RARγ‐dependent. A431 cells were transfected with RARγ siRNA (A) or Flag‐RARγ expression plasmid (B) for 24 hours before exposing to 1 μM 3‐Cl‐AHPC or 1 μM ATRA for 12 hours. Cell lysates were analysed by Western blotting using M24, anti‐RARγ and anti‐Flag antibodies for detecting matriptase/HAI‐1 complex, RARγ and Flag‐RARγ

3‐Cl‐AHPC has been identified as a cognate ligand of nuclear receptor RARγ. It possesses both RARγ‐ dependent and ‐independent biological activities.^31^, ^41^ We therefore explored whether 3‐Cl‐AHPC effect on matriptase was RARγ‐dependent. We first compared 3‐Cl‐AHPC with another RARγ ligand all‐trans retinoic acid (ATRA). Different from 3‐Cl‐AHPC strong induction of matriptase/HAI‐1 complex, ATRA did not show obvious effect at the same condition (Figure 6). Moreover, 3‐Cl‐AHPC strong induction of matriptase/HAI‐1 complex was not significantly affected by either RARγ down‐expression using siRNA (Figure 6A) or RARγ overexpression using expression plasmid (Figure 6B). Thus, the induction of matriptase/HAI‐1 complex formation by 3‐Cl‐AHPC may not be RARγ‐dependent.

## 4 DISCUSSION

The oncogenic ability of matriptase is manifested in a variety of cancer types, rendering matriptase an attractive target for cancer therapy.^3^, ^4^ Small molecules can be designed to inhibit matriptase proteolytic activity via direct binding to the catalytic sites or preventing its cleaving maturation.^28^, ^42^, ^43^, ^44^ However, we report here a distinct strategy for small molecule design to blocking matriptase activity via inducing matriptase/HAI‐1 complex.

**Figure 7:**
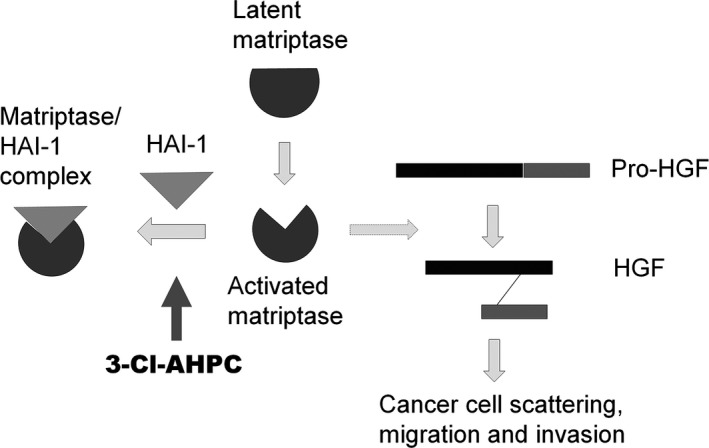
Model of 3‐Cl‐AHPC's effect on matriptase/HAI‐1 complex formation and pro‐HGF maturation. 3‐Cl‐AHPC inhibits matriptase activity via inducing the activated matriptase‐forming complex with HAI‐1, leading to down‐regulation of matriptase‐mediated pro‐HGF maturation and pro‐HGF‐induced cancer cell scattering, migration and invasion

The activity of matriptase largely depends on the balance of zymogen activation and proteolytic activity inhibition. The complex formation of matriptase/HAI‐1 indicates that matriptase is activated and/or the activity of matriptase is quenched by HAI‐1.^2^, ^45^ EGF, DHT and suramin activate matriptase followed by matriptase/HAI‐1 complex induction.^6^, ^8^, ^36^ 3‐Cl‐AHPC also induces matriptase/HAI‐1 complex formation, but it inhibits rather than activates matriptase (Figures 1 and 2), indicating that 3‐Cl‐AHPC induction of matriptase/HAI‐1 is not because of matriptase activation. With M69 antibody, specifically recognizing the activated but not the latent matriptase,^34^, ^35^ we find 3‐Cl‐AHPC time‐ and dose‐dependently induces the matriptase/HAI‐1 complex formation and reduces free activated matriptase level simultaneously (Figure 1A and B). Thus, 3‐Cl‐AHPC increases the complex most likely through enhancing the association of activated matriptase and HAI‐1 rather than activating matriptase as suramin (Figure 7). Owing to the high matriptase/HAI‐1 ratio and high matriptase activation in cancer cells,^3^, ^4^ activated matriptase is not saturated through forming complex with HAI‐1, leading to relatively high amount of activated matriptase in cancer cells. It is hypothesized that matriptase is only activated for a brief interval at the cell surface prior to its binding and inactivation by HAI‐1. The enhancement of activated matriptase association with HAI‐1 by 3‐Cl‐AHPC should not only reduce the amount of activated matriptase but also shorten the interval of the activated matriptase at the cell surface, leading to down‐regulation of matriptase activity in cancer cells.

HAI‐1 is critical to control matriptase proteolytic activity.^20^ However, HAI‐1 expression is reduced in many cancer types, which is implicated in the progression of cancer and is associated with a worse prognosis in cancer patients. The insufficient HAI‐1 leads to the higher matriptase/HAI‐1 ratio and aberrant matriptase activity.^3^, ^4^ Engineered expression of HAI‐1 dramatically inhibits the invasion and migration of cervical, endometrial and uterine cancer cells in vitro.^1^, ^2^, ^3^, ^4^ However, the approach to increase HAI‐1 expression in vivo or in patient is not easy to achieve. Even in a relatively low expression level, there is still free HAI‐1 available for binding to activated matriptase in cancer cells (Figures 1 and 2). 3‐Cl‐AHPC may enhance the binding affinity of HAI‐1 to activated matriptase, enabling cancer cell to use relatively limited amount of HAI‐1 to block matriptase activity. Thus, 3‐Cl‐AHPC could increase the blocking efficiency of HAI‐1 (Figure 7), suggesting a useful strategy to block matriptase activity. Besides matriptase, HAI‐1 also binds to and inhibits a variety of serine proteases including of hepsin, plasmin, prostasin, TMPRSS13 and HAT. It is of great interests to investigate whether 3‐Cl‐AHPC or other compounds could promote HAI‐1 forming complex with these serine proteases followed by inhibiting their activity, benefiting to the treatment of cancer and other diseases.

Matriptase shedding requires proteolytic cleavage distinct from but tightly coupled with its maturation cleavages.^46^ In general, shedding from cell membrane increases the duration of matriptase activation and the accessibility of matriptase to its substrates. The shed matriptase contains the catalytic ectodomain and in tumour microenvironment may more readily activate oncogenic substrates and change the ECM contributing to tumour progression.^7^, ^46^, ^47^ 3‐Cl‐AHPC also induces matriptase shedding (Figure 2B), but it is not certain whether 3‐Cl‐AHPC‐induced matriptase/HAI‐1 complex formation and shedding are correlative or not. 3‐Cl‐AHPC also induces matriptase/HAI‐1 complex in the conditioned medium (Figure 2B), but it also remains unclear whether the complex induction occurs before or after shedding. Important is that 3‐Cl‐AHPC‐induced shedding does not increase the matriptase activity in the conditioned medium (Figure 2C and D). Similarly, curcumin potently induces matriptase shedding without increasing shed matriptase activity.^6^ The difference is that 3‐Cl‐AHPC enhances but curcumin inhibits matriptase/HAI‐1 complex formation at the plasma membrane, implying different mechanisms of two compounds on inhibiting matriptase activity.

Cell first synthesizes pro‐HGF secreting to ECM. Matriptase is involved in the maturation of pro‐HGF via cleavage.^48^ Matriptase fails to promote tumour progression in epidermal‐deficient c‐Met mice, which indicates that pro‐HGF‐cleaving activation is an essential step by which matriptase exerts cancer promotion.^2^, ^3^, ^4^, ^48^ 3‐Cl‐AHPC inhibits pro‐HGF‐induced signal transduction and cell scattering (Figures 4 and 5). Importantly, the function of 3‐Cl‐AHPC is matriptase dependent, because suppression of matriptase expression inhibits both pro‐HGF signal pathway and 3‐Cl‐AHPC effect (Figures 4D and 5), which is consistent with 3‐Cl‐AHPC inhibiting pro‐HGF maturation through matriptase‐mediated cleavage (Figure 4A). Pro‐HGF in the cell medium could be cleaved to mature by cell surface matriptase and/or shedding matriptase. 3‐Cl‐AHPC inhibition of pro‐HGF signal also verifies that the net activity of matriptase at the plasma membrane and in the conditioned medium is inhibited by 3‐Cl‐AHPC, which is consistent with our proteolytic activity assay (Figure 2). Therefore, 3‐Cl‐AHPC is able to block matriptase/pro‐HGF/c‐Met cascade via down‐regulating matriptase activity to inhibit cancer cell scattering, migration and invasion (Figure 7).

DHT robustly activates matriptase and induces matriptase/HAI‐1 complex formation as well as shedding in an AR‐dependent manner.^7^ DHT has been shown to activate AR to promote TMPRSS2 expression, followed by induction of TMPRSS2‐mediated matriptase cleavage and activation.^8^ 3‐Cl‐AHPC is an analogue of retinoid AHPN and a ligand of RARγ. It binds to RARγ but does not activate RARγ transcriptional activity.^30^ It has been shown potent anti‐cancer effects in a RARγ‐independent manner.^30^ Similarly, we find that the anti‐matriptase activity of 3‐Cl‐AHPC may not depend on RARγ either (Figure 6). Thus, 3‐Cl‐AHPC has other targets in cells to mediate its promotion of matriptase/HAI‐1 complex formation. The orphan nuclear receptor small heterodimer partner (SHP) has been identified as a target for 3‐Cl‐AHPC to exert its anti‐cancer effect.^49^, ^50^ However, whether 3‐Cl‐AHPC binding to SHP is responsible for its induction of matriptase/HAI‐1 complex remains to be clarified. The robust anti‐cancer effect of 3‐Cl‐AHPC on a variety of cancer types has been widely acknowledged, however, the underlying mechanism is still elusive. Here, we show that inhibiting matriptase also contributes to 3‐Cl‐AHPC anti‐cancer effect, which provides a new direction to optimize 3‐Cl‐AHPC for cancer treatment.

In all, our research provides a new mechanism underlying 3‐Cl‐AHPC anti‐cancer effect and a new strategy to antagonize matriptase activity through enhancing matriptase/HAI‐1 complex formation using small molecules.

## CONFLICT OF INTEREST

The authors confirm that there are no conflicts of interest.
